# Identifying and Implementing Best Practices for Including Persons Living With Dementia in Research

**DOI:** 10.1093/geroni/igaf122.587

**Published:** 2025-12-31

**Authors:** Brianna Morgan, Elizabeth Carpenter-Song, Alejandra Martinez, Lisa Mistler, Salar Khaleghzadegan, Wambui Onsando, Joshua Chodosh, Paul Barr

**Affiliations:** New York University Langone Health, New York, New York, United States; Dartmouth College, Hanover, New Hampshire, United States; The Dartmouth Institute for Health Policy and Clinical Practice, Lebanon, New Hampshire, United States; Dartmouth College, Hanover, New Hampshire, United States; Dartmouth College, Hanover, New Hampshire, United States; The Dartmouth Institute for Health Policy and Clinical Practice, Lebanon, New Hampshire, United States; NYU Langone, New York City, New York, United States; The Dartmouth Institute for Health Policy and Clinical Practice, Lebanon, New Hampshire, United States

## Abstract

Including persons living with dementia (PLwD) in research is increasingly a priority for professional organizations (e.g., Gerontological Society of America) and national research strategies (e.g., National Plan to Address Alzheimer’s). However, PLwD have unique barriers to effective communication, including memory impairment, word-finding difficulty, and executive dysfunction, limiting their participation. Further, few research studies explicitly describe practices for facilitating the participation of PLwD in research, henceforth termed “inclusive practices”. Our aim was to develop and pilot an inclusive practice guide that allowed PLwD to share their perspectives in a study on the barriers and enablers to interpersonal communication in triadic healthcare visits (i.e., PLwD, care partner, healthcare provider). We identified best practices for including PLwD in research from the literature, which directed our interview guide and training manual development. We then mapped the interview questions onto the Capability-Opportunity-Motivation model of Behavior (COM-B) theoretical framework to understand interpersonal communication and the component(s) to target for change. We also translated the guide into Spanish. Using purposive sampling to identify participants who could provide feedback on the interview guide, we pilot-tested the interview guide with seven participants living with dementia, five in English (in Lebanon, New Hampshire) and two in Spanish (in New York City, New York). Participants found the interview guide acceptable and provided suggestions for improvement. The final interview guide generated robust responses to the research question without causing distress. Explicitly incorporating inclusive practices for including PLwD research is an essential step in soliciting the important perspectives of PLwD.

